# Genetic and Clinical Characteristics of Patients with Vitamin D Dependent Rickets Type 1A

**DOI:** 10.4274/jcrpe.galenos.2018.2018.0121

**Published:** 2019-02-20

**Authors:** Fatma Dursun, Gamze Özgürhan, Heves Kırmızıbekmez, Ece Keskin, Bülent Hacıhamdioğlu

**Affiliations:** 1 Ümraniye Training and Research Hospital, Clinic of Pediatric Endocrinology, İstanbul, Turkey; 2 Süleymaniye Maternity and Children’s Training and Research Hospital, Clinic of Paediatrics, İstanbul, Turkey; 3 Süleymaniye Maternity and Children’s Training and Research Hospital, Clinic of Medical Genetic, İstanbul, Turkey; 4 İstinye University Faculty of Medicine, Department of Pediatric Endocrinology, İstanbul, Turkey

**Keywords:** Vitamin D, vitamin D dependent rickets, CYP27B1 gene, 1α hydroxylase

## Abstract

**Objective:**

Vitamin D dependent rickets type 1A (VDDR1A) is an autosomal recessive disorder caused by mutations in the 1α-hydroxylase gene (*CYB27B1*). As it may be confused with nutritional rickets and hypophosphatemic rickets, genetic analysis is important for making a correct diagnosis.

**Methods:**

We analysed genomic DNA from 11 patients from eight different Turkish families. The patients were recruited for our studies if they presented with a diagnosis of VDDR.

**Results:**

The mean ± standard deviation age at diagnosis was 13.1±7.4 months. Seven patients had mild hypocalcemia at presentation while four patients had normal calcium concentrations. All patients underwent *CYP27B1* gene analysis. The most prevalent mutation was the c.195 + 2T>G splice donor site mutation, affecting five out of 11 patients with VDDR1A. Two patients from the fourth family were compound heterozygous for c.195 + 2T>G and c.195 + 2 T>A in intron-1. Two patients, from different families, were homozygous for a previously reported duplication mutation in exon 8 (1319_1325dupCCCACCC, Phe443Profs*24). One patient had a homozygous splice site mutation in intron 7 (c.1215 + 2 T>A) and one patient had a homozygous mutation in exon 9 (c.1474 C>T).

**Conclusion:**

Intron-1 mutation was the most common mutation, as previously reported. All patients carrying that mutation were from same city of origin suggesting a “founder” or a “common ancestor” effect. VDDR1A should definitely be considered when a patient with signs of rickets has a normal 25-OHD level or when there is unresponsiveness to vitamin D treatment.

**What is already known on this topic?**

Although vitamin D dependent rickets type 1A (VDDR1A) is a rare disease, it is relatively more common in Turkey. Thus far intron-1 mutations have only been reported from Turkey. Intron-1 mutations have been reported to be associated with milder clinical findings. Clinical and laboratory findings can overlap with other types of rickets. Serum 1,25-dihydroxyvitamin D levels are usually reported to be low in cases of VDDR1A.

**What this study adds?**

Patients with intron-1 mutations can present with clinical findings of variable severity. We also found that the concentrations of 1,25-dihydroxyvitamin D levels may be within inappropriately normal ranges in genetically proven vitamin D dependent rickets type 1A and lead to diagnostic confusion.

## Introduction

Vitamin D (calciferol) comprises two biologically inactive, fat-soluble pro-hormones. The first is ergocalciferol (vitamin D2), derived from ergosterol after ultraviolet (UV) light exposure and the second is cholecalciferol (vitamin D3), derived from animal tissues and 7-dehydrocholesterol, formed in human skin by the action of UV rays in sunlight (1). Both forms need a two-step hydroxylation at the 25^th^ and 1^st^ carbons for activation. The first step occurs in the liver, where vitamin D is hydroxylated to 25-hydroxyvitamin D (25-OHD) by hepatic 25-hydroxylase. The second step occurs mainly in the kidney, where 25-OHD is further hydroxylated by the mitochondrial vitamin D 1α-hydroxylase to the biologically active hormone 1,25-dihydroxyvitamin D (1,25-OH_2_D), which binds to its nuclear receptor and exerts its biological activities (1,2,3). The biologically active 1,25-OH_2_D plays a central role in calcium homeostasis and bone metabolism and also has a significant influence on cell proliferation and differentiation of a variety of tissues (1,3,4). The renal synthesis of 1,25-OH_2_D from its precursor 25-OHD is a rate-limiting step and is tightly regulated by exisiting serum concentrations of 1,25-OH_2_D, parathyroid hormone (PTH), fibroblast growth factor-23 (FGF-23), calcium and phosphate concentrations, with renal 1α-hydroxylase being stimulated by PTH, hypophosphatemia, or hypocalcaemia and inhibited by FGF-23 (4).

Four rare genetic errors of vitamin D metabolism that can cause rickets have been described. The first one involves 1α-hydroxylase deficiency, which is also described as vitamin D dependent rickets type 1A (VDDR1A). A selective mutation in *CYP2R1* gene, which leads to 25-hydroxylase deficiency, is called type 1B (VDDR1B). This second type involves a defective vitamin D receptor (VDR), resulting in vitamin D resistant rickets (VDRR), also known as VDDR type 2A (VDDR2A). VDDR2B is an unusual form of rickets due to abnormal expression of a hormone response element-binding protein that interferes with normal function of VDR (5,6,7,8).

VDDR1A is an autosomal recessive disorder caused by mutations in the 25-OHD 1α-hydroxylase gene (*CYB27B1*). *CYB27B1* is composed of nine exons and is approximately 5 Mb in size. The gene has been mapped to the chromosomal region 12q14.1 (9,10,11,12). Clinically, VDDR1A is characterized by hypotonia, muscle weakness, inability to walk, growth failure and radiographic findings of rickets. Typical laboratory findings are hypocalcaemia, elevated serum levels of alkaline phosphatase (ALP) and of PTH with low or normal levels of 1,25-OH_2_D despite normal or increased concentrations of 25-OHD (9,13). Patients with VDDR1 may present with aminoaciduria and hyperchloremic acidosis (3).

To date, over 100 patients with 78 mutations have been identified in the *CYP27B1* gene in patients from multiple ethnic groups. These mutations span all exons of the gene and mostly include missense and nonsense changes, along with splice site changes, insertions, deletions and duplications [Human Gene Mutation Database (HGMD), http://www.hgmd.cf.ac.uk/ac/index.php] (14). Mutations in *CYP27B1* lead to a loss of 1α-hydroxylase activity and require treatment with calcitriol to normalize the clinical and laboratory abnormalities (15).

In the present study, we report 11 patients with VDDR1A from eight unrelated Turkish families. The most prevalent mutation was the c.195 + 2T>G splice donor site mutation, affecting five out of 11 patients with VDDR1A. Clinical findings of patients were examined in detail and genotype-phenotype correlations were evaluated.

## Methods

We analyzed genomic DNA in 11 patients from eight different Turkish families. In five of these families, the parents were consanguineous. The study was approved by the University of Health Sciences Ümraniye Training and Research Hospital Clinical Research Ethical Committee (approved number: 19/01/2018-2926). Informed consent was obtained from patients and/or families.

Eleven patients had the clinical findings of rickets including X-bain deformity or bowed leg, chest rosary, Harrison’s groove, frontal bossing, widening of the wrist, growth retardation, hypotonia and inability to walk together with hypocalcaemic seizures. The patients also had biochemical features suggestive of rickets such as hypophosphatemia, hypo- or normocalcemia, elevated PTH and ALP, normal or high 25-OHD levels and low or normal 1,25-OH_2_D levels. Wrist and knee radiographs of all patients demonstrated widened epiphyses and metaphyseal cupping and fraying. Differentiation of nutritional rickets and VDDR1A was made by normal/high 25-OHD levels, low/inappropriately normal 1,25-OH_2_ D levels and improvement in the clinical, biochemical and radiological findings of rickets after replacement with calcitriol. All patients received calcitriol and patients with hypocalcaemia received calcium replacement. Calcitriol was started at a dose of 1-1.5 mcg/day, twice daily. Subsequently the calcitriol dose was titrated according to the results of biochemical analyses. The aims of the treatment were to achieve normocalcemia, to maintain PTH levels within normal limits and to avoid hypercalciuria.

### Targeted Second Generation Sequence Analysis

DNA was isolated from a 200 microlitre peripheral blood sample using QIAamp DNA Blood Mini QIAcube Kit and QIAcube device (QIAGEN, Hilden, Germany). Then, the exons of the *CYP27B1* gene were amplified for targeted sequencing. Amplification was controlled with agarose gel electrophoresis technique. Sequencing was carried out using Illumina MiSeq NGS System (Illumina Inc., San Diego, CA, USA) and the Miseq Reagent Kit V3 (600 cycles) from the same manufacturer. The readings were aligned with human genome 19 genomic sequence and compared.

### Sanger Sequencing

10 mL venous blood sample was taken from each patient into EDTA tubes. DNA isolation was performed using the QIAamp DNA Mini QIAcube Kit from the peripheral blood. The Primer design included *CYP27B1* gene exons and close introns (Table 1). The products of polymerase chain reaction (PCR) [94 °C-5 min (95 °C-30 sec - 60 °C-30 sec - 72 °C 30 sec) x 34, 72 °C-5 min] with the primers, also shown in Table 1, were checked on a 2% agarose gel. After the amplification of correct gene regions, purification of PCR products was made by maintenance for 15 minutes at 37 °C (enzyme activation temperature) and 15 minutes at 80 °C (enzyme inactivation temperature) in the thermal cycler using ExoSAP enzyme. After purification, the primer and the cleaned template DNA were added to the PCR solution, using “The Big Dye Ready Reaction Mix Sequencing Kit” (Applied Biosystems^®^ Big Dye^®^, Foster City, Calif., USA) and the PCR reaction was started. The purification process was repeated after the PCR sequencing for the removal of uncoupled dideoxynucleotide triphosphates in the solution. Sanger sequencing of the purified samples was performed on the ABI 3130 XL (Applied Biosystems^®^ 3130 Genetic Analyzers, Foster City, Calif., USA) capillary sequencing device. The obtained data were analysed by Applied Biosystems SeqScape^®^ Software (Calif., USA) analysis program.

### Data Analysis

Sequenced data were analyzed with the Genomize Variant Analysis Program (NHLBI GO Exome Sequencing Project, Seattle, USA) and Integrative Genomics Viewer (1000 Genomes Project, Calif., USA). The homozygote or compound heterozygote variants in the databases such as National Center for Biotechnology Information, HGMD, and Clinvar were primarily selected for data filtering. The effects of mutations on protein structures were tested with various *in silico* prediction tools, particularly Mutation Taster (16), PolyPhen-2 (17), and Sorting Tolerant From Intolerant (18).

### Statistical Analysis

Statistical analysis was performed using IBM SPSS 21.0 for windows statistical software (IBM Inc., Chicago, Ill., USA). The data were presented as mean ± standard deviation (SD) (ranges).

## Results

Among patients diagnosed with VDRR1A, six were males and five were females, from eight families. Clinical presentation and laboratory findings of the patients are summarized in Table 2. The mean age at diagnosis was 13.1±7.4 months. Seven patients had mild hypocalcemia at presentation while four patients had normal calcium levels. Five of eight families had consanguineous marriages. The two families that were not consanguineous were from the same city.

**Figure 1:**
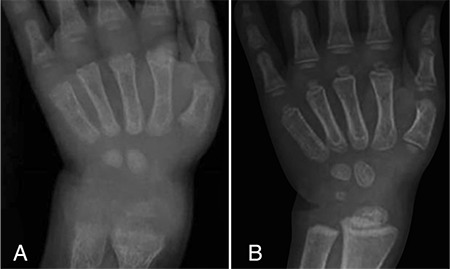
X-rays of this patient before (A) and at the 6th month of calcitriol treatment (B) (A) Abnormal cupping, widening and fraying of the metaphyses consistent with rickets. (B) Recovery of cupping and fraying, and a provisional calcification zone suggesting healing rickets

All patients had clinical and laboratory features of rickets at the time of diagnosis. All patients had low levels of phosphorus with quite high levels of PTH and ALP levels (see Table 2). Five patients had fairly high levels of 25-OHD due to being formerly diagnosed with nutritional rickets and treated with vitamin D. Levels of 1,25-OH_2_D, on the other hand, were normal in three patients. One patient was previously followed for hypophosphatemic rickets and treated with calcitriol and phosphate. When he was diagnosed with VDDR1A, he had elevated PTH levels and typical radiological findings of rickets (Figure 1). 

After the definitive diagnosis of VDDR1A all patients received calcitriol treatment. The duration of treatment with calcitriol ranged between six months and seven years. Biochemical improvement with treatment occurred within a period ranging between four and 12 months.

**Figure 2:**
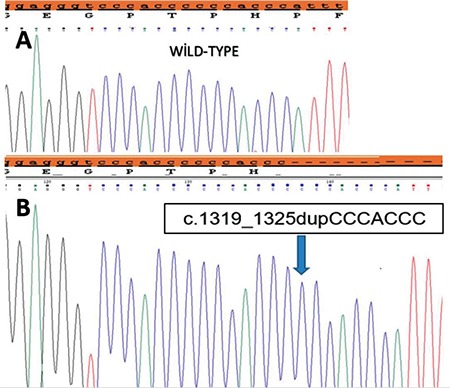
A) Wild type sequence of exon 8 in CYP27B1 gene. B) Sequencing analysis of the CYP27B1 gene exon 8 showing the homozygous mutation (1319_1325dupCCCACCC, Phe443Profs*24)

All patients underwent *CYP27B1* gene analysis (Table 3). The most prevalent mutation was the c.195 + 2T>G splice donor site homozygous mutation, affecting five out of 11 patients with VDDR1A. Two patients from family-4 had a compound heterozygous mutation for c.195 + 2T>G and c.195 + 2 T>A in intron-1. Two patients from different families had homozygous duplication mutation in exon 8 (1319_1325dupCCCACCC, Phe443Profs*24), which has been previously reported (Figure 2). A homozygous c.1215 + 2T>A mutation in the splice donor site of intron-7 was found in one patient and one patient was found to have a homozygous mutation in exon 9 (c.1474 C>T).

## Discussion

In the present study, we report the clinical, biochemical and genetic analysis of 11 patients with VDDR1A. We identified five previously reported mutations. The most prevalent mutation was the c.195 + 2T>G splice donor site mutation. Five patients from two different families had this mutation as homozygous and two patients from the same family had hemizygous inheritance as a part of compound heterozygous mutation. Durmaz et al (19) reported this mutation for the first time in a Turkish patient. Currently the c.195 + 2T>G homozygous mutation in intron-1 is present in a total of 20 patients including the patients described herein, all reported from Turkey (4,9,19). These patients were homozygous for the previously described splice donor site mutation c.195 + 2T>G, where a thiamine is substituted for a guanine in the second nucleotide of intron-1. Since this mutation is common in Turkish patients and has not been reported in other ethnic groups, it may be unique, representing a ‘founder’ or “common ancestor” effect, given the high rates of consanguinity. Although it has not been reported in other publications, all patients in the study by Tahir et al (9) were living in Diyarbakır or neighbouring provinces; all of our patients carrying that mutation were from Batman, which is geographically very close to Diyarbakır.

While Tahir et al (9) reported that patients with intron-1 mutation had a milder clinical presentation, Demir et al (4) reported that the most severe form of the disease occurred in a patient with intron-1 mutation thus the phenotype may be variable and a larger evidence base would be necessary to determine the genotype/phenotype relationship more clearly. We could not identify any relationship between genotype and phenotype although our series adds to the existing evidence. All patients in the literature who had an intron-1 mutation had delayed walking and bowed legs at admission. While four of our patients were also affected thus, another patient presented with hypotonia. Although 4 of 5 patients with intron-1 mutation had a height below -2 SD, patients with other mutations also had short stature. 

We had only one patient presenting at the age of 11 months with a hypocalcaemic convulsion. Hypocalcaemic convulsion has also been reported rarely by other studies from Turkey. Tahir et al (9) reported hypocalcaemic convulsion in five of 22 patients; Demir et al (4) in 4 of 8 patients; and Durmaz et al (19) in two of seven patients. Kim et al (20) reported that 4 of their 10 patients presented with hypocalcaemic convulsion. Edouard et al (21) reported that the admission symptom was hypocalcaemic convulsion in 4 of 21 pediatric patients. Since these patients had blood calcium levels that were in the lower limit of the normal range, hypocalcemic convulsion was not frequently encountered.

The clinical presentations of patients with VDDR1A could lead to a misdiagnosis of nutritional rickets or hypophosphatemic rickets, which can be differentiated from hypophosphatemic rickets by a high PTH level and from nutritional rickets by a normal 25-OHD level. The hypophosphatemia in VDDR1A is a result of elevated PTH and renal excretion of phosphate. The clinical and laboratory features of VDDR1A are very similar to nutritional rickets although the differential diagnosis can be made by a low or inappropriately normal 1, 25-OH_2_D level and unresponsiveness to vitamin D treatment. In our study, six patients had also had long-term therapy with vitamin D because of an initial diagnosis of nutritional rickets and they had extremely high 25-OHD levels. Four patients had normal calcium levels and one of them had been followed with hypophosphatemic rickets. There are a few patients with normal 1,25-OH_2_D levels diagnosed with VDDR1A in the literature (4,8). In fact, the expected 1,25-OH_2_D levels in 1a-hydroxylase deficiency are low and inappropriately normal 1,25-OH_2_D levels also indicate that the enzyme activity is insufficient. Recently, Nishikawa et al (22) reported that liver mitochondrial *CYP27A1* can catalyse 1α-hydroxylation of 25-OHD. A small increase in serum 1,25-OH_2_D concentration has been observed in *CYP27B1* knockout mice after being given high dietary vitamin D, suggesting a conversion from 25-OHD to 1,25-OH_2_D by a non-*CYP27B1* enzyme. Three of eleven patients in our study had normal 1,25-OH_2_D levels and there was a history of high dose vitamin D intake in two of these three patients. In these patients, conversion from 25-OHD to 1,25-OH_2_D by a non-*CYP27B1* enzyme may have contributed to the normal 1,25-OH_2_D level. 

Maternal 1,25-OH_2_D does not cross the fetoplacental barrier (21,23). 1,25-OH_2_D increases 2-3 fold in the first weeks of pregnancy when maternal 25-OHD crosses the placental barrier. The rise in circulating 1,25-OH_2_D concentrations in the mother facilitates optimal *in utero* bone development by attaining a positive calcium balance (24). Edouard et al (21) reported that, unlike patients with severe vitamin D deficiency who can present within the first six months of life, none of the VDDR1A patients were symptomatic before the age of six months. Indeed, the infant who was diagnosed with VDDR1A at the age of one month had a low serum 1,25-OH_2_D and a positive *CYP27B1* sequencing result but did not have any clinical or radiological signs of rickets (21). This indicates that 1,25-OH_2_D is not critical for mineral ion homeostasis and growth plate mineralization in the first months of life owing to *in utero* positive calcium balance in these patients. All patients in this study group were aged 6-months or older at admission.

Generally, a good response to treatment with alfacalcidol or calcitriol (10-400 ng/kg/day) is expected in cases with VDDR1A (4,21). Calcitriol dose was tailored based on biochemical and clinical findings. Edouard et al (21) indicated short and long-term outcomes of calcitriol treatment in their patients. They started calcitriol treatment at a dose of 1.0 µg/day, given in two doses of 0.5 µg. Treatment with calcitriol resulted in the normalization of biochemical parameters within three months. The aims of the treatment were to achieve normocalcemia, to maintain PTH levels within normal limits and to avoid hypercalciuria. Our patients had not reached their final height and their treatment durations ranged between six months and seven years. Improvement of biochemical parameters occurred somewhat later than previously reported at between four and 12 months.

### Study Limitations

The main limitation of our study is the relatively small number of patients.

## Conclusion

Although VDDR1A is a rare disease, it is more common in Turkey where autosomal recessive disorders are common. In this study, we evaluated the genetic and clinical features of 11 patients with the diagnosis of VDDR1A. Intron-1 mutation was the most common mutation, as in the previous studies, and all patients carrying this mutation were from the same city of origin, suggesting a “founder” or a “common ancestor” effect. As it may be confused with nutritional rickets and hypophosphatemic rickets, genetic analysis is important for making a correct diagnosis. VDDR1A should be considered when a patient with signs of rickets has a normal 25-OHD level or when there is unresponsiveness to vitamin D treatment. We should emphasize that concentrations of 1,25-OH_2_D levels can be within normal ranges in patients with VDDR1A and this may lead to diagnostic confusion.

## Figures and Tables

**Table 1:**
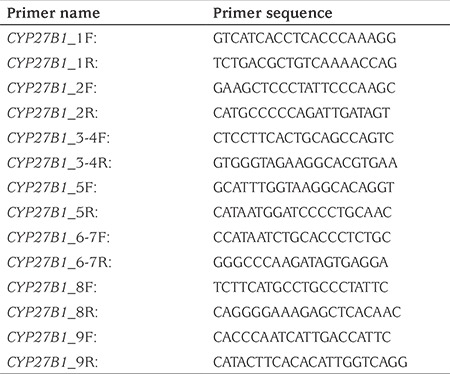
List of primers used for polymerase chain reaction amplification of the nine coding exons of *CYP27B1* gene

**Table 2:**
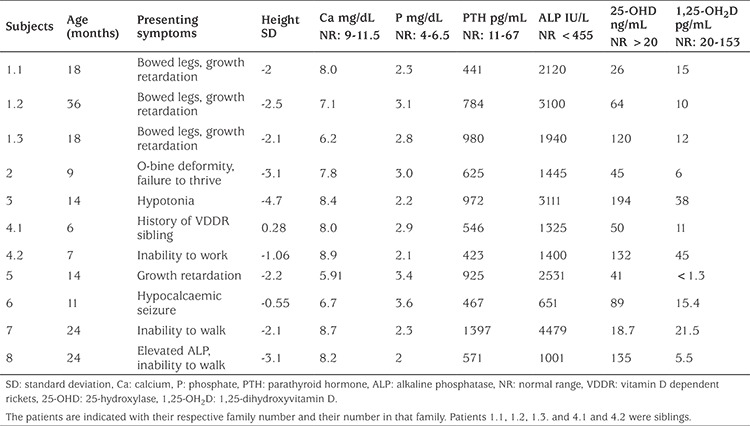
Clinical and laboratory findings of 11 patients with vitamin D dependent rickets type 1A from 8 families

**Table 3:**
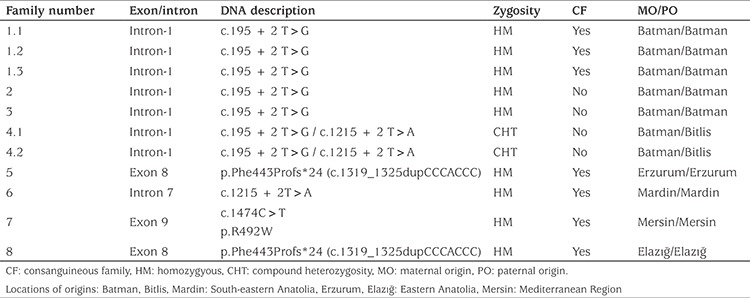
Characteristics of the mutations detected in 11 patients with vitamin D dependent rickets type 1A from eight families
